# The Wandering Knife: A Case Report

**DOI:** 10.7759/cureus.44575

**Published:** 2023-09-02

**Authors:** Anamika Nepal, Ashish P Rajbhandari

**Affiliations:** 1 Internal Medicine, Shankarapur Hospital, Kathmandu, NPL; 2 General Surgery, Shankarapur Hospital, Kathmandu, NPL

**Keywords:** resource-limited setting, surgical case report, retained foreign bodies, sharp penetrating foreign bodies, laparotomy, foreign body retrieval, abdominal stab wounds

## Abstract

Stab injuries to the abdomen have become a common occurrence, though retained objects are rare. A 22-year-old male presented with a left lower abdominal discomfort the next day after having a stab in the right hypochondrium. He was hemodynamically stable, with no signs of peritonitis. Abdominal X-ray revealed a 15 cm long knife blade in the left lower abdomen. He underwent a laparotomy for the removal of the knife blade located inside the peritoneal cavity in his left iliac region without any injury to the surrounding viscera.

## Introduction

Stab injuries to the abdomen are commonly encountered in surgical practice [1]. However, less is reported about free-floating sharp foreign bodies in the abdominal cavity causing no major visceral injury. It is a potentially life-threatening condition to have a knife blade in proximity to significantly vital visceral organs in the abdomen. These kinds of cases require a careful clinical and radiological assessment before proceeding with definitive management [2]. Here, we present a case of a 22-year-old male who was stabbed with a knife one day prior to presentation and was initially managed with the closure of the abdominal wound without exploration, leaving the blade of the knife inside the abdominal cavity. He presented to the hospital after having continued abdominal pain. Imaging revealed a free-floating knife blade in the abdominal cavity, which was subsequently removed by laparotomy. The objective of this case report is to report a rare case of a sharp foreign body retained in the abdominal cavity without any significant visceral injury and review our experience with managing such a case.

## Case presentation

**Figure 1:**
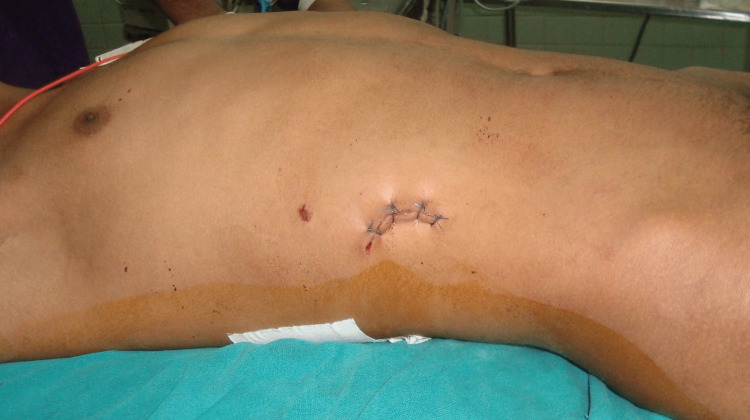
Abdomen with suture wound on the right hypochondrium

A 22-year-old male arrived in the emergency department (ED) of our hospital, which is a secondary-level hospital in Nepal, complaining of mild continuous left lower abdominal pain of one-day duration. He denied abdominal distension, nausea, vomiting, diarrhea, or constipation. He was alert, oriented, and hemodynamically stable on examination. His abdominal examination revealed a sutured wound on the right hypochondrium (Figure 1). There was tenderness over the left iliac region, without guarding or rigidity and normal bowel sounds. Upon retaking the history, he said he was stabbed by a knife in a fight a day back. It caused a penetrating wound in the right hypochondrium with an unknown amount of blood loss. He was taken to a local medical shop where the wound was sutured by an auxiliary health worker, but no documentation of injury or procedure was done. Since there was no direct visualization of any retained foreign body, nobody suspected there could be any retained foreign body inside the abdominal cavity. He was sent home from there as the wound was closed well. He did not remember much about other events as he was in an intoxicated state with alcohol when the incident happened.

**Figure 2:**
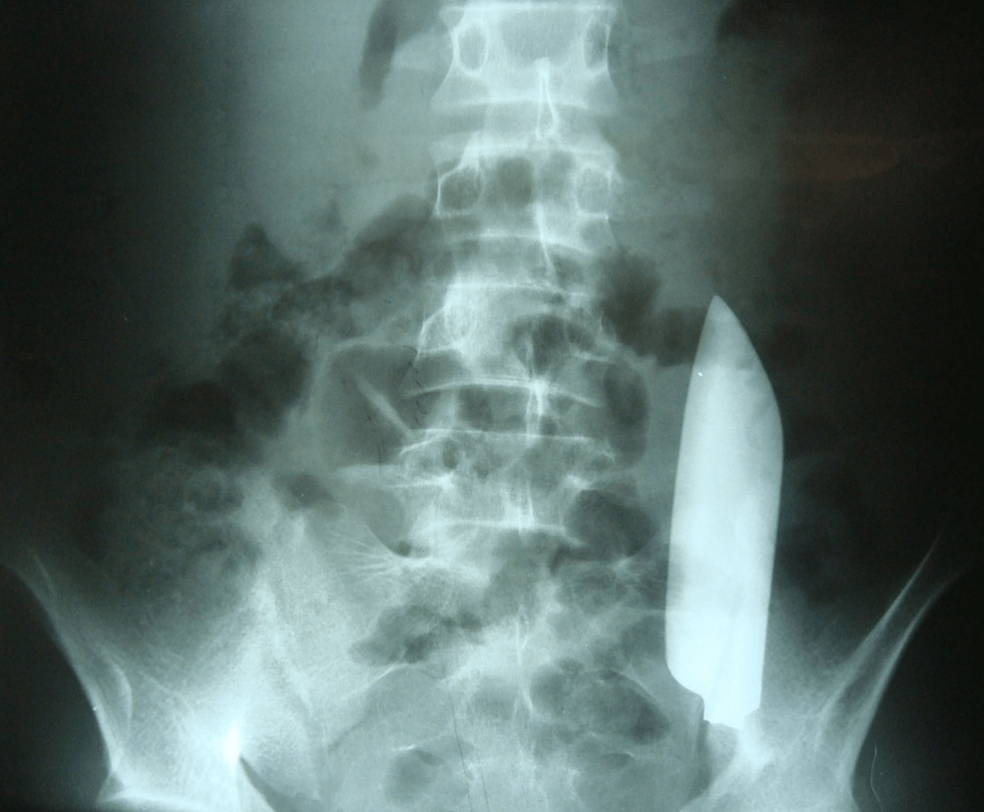
Abdominal X-ray in the posteroanterior view showing a free-floating metallic knife blade in the abdomen

His preliminary labs, including complete blood count, comprehensive metabolic panel, urinalysis, and coagulation panel, were within normal limits. His abdominal X-ray revealed a 15 cm long knife blade in the left lower abdomen (Figure 2).

**Figure 3:**
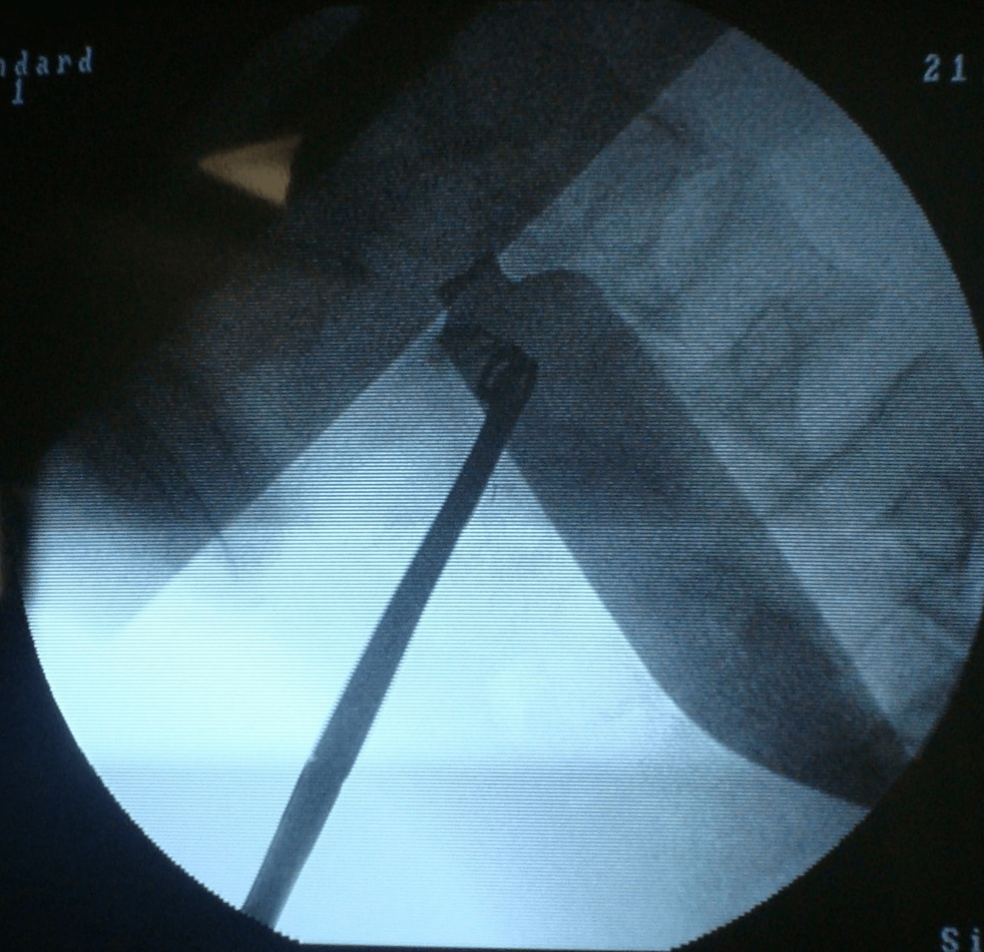
Fluoroscopy showing the knife blade in the abdomen

**Figure 4:**
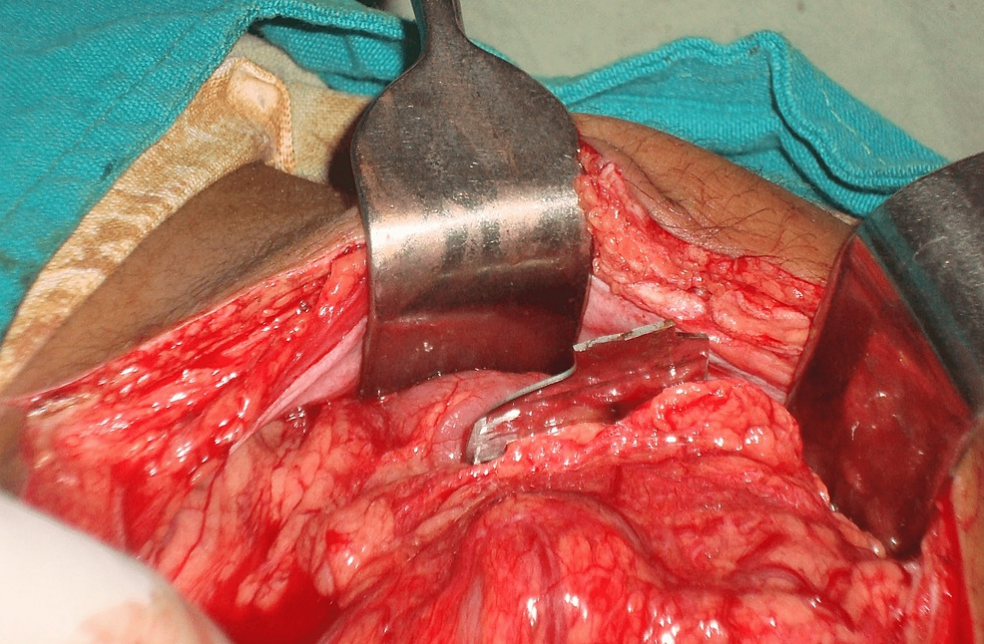
Intraoperative photo during retrieval of the knife blade from the abdominal cavity

**Figure 5:**
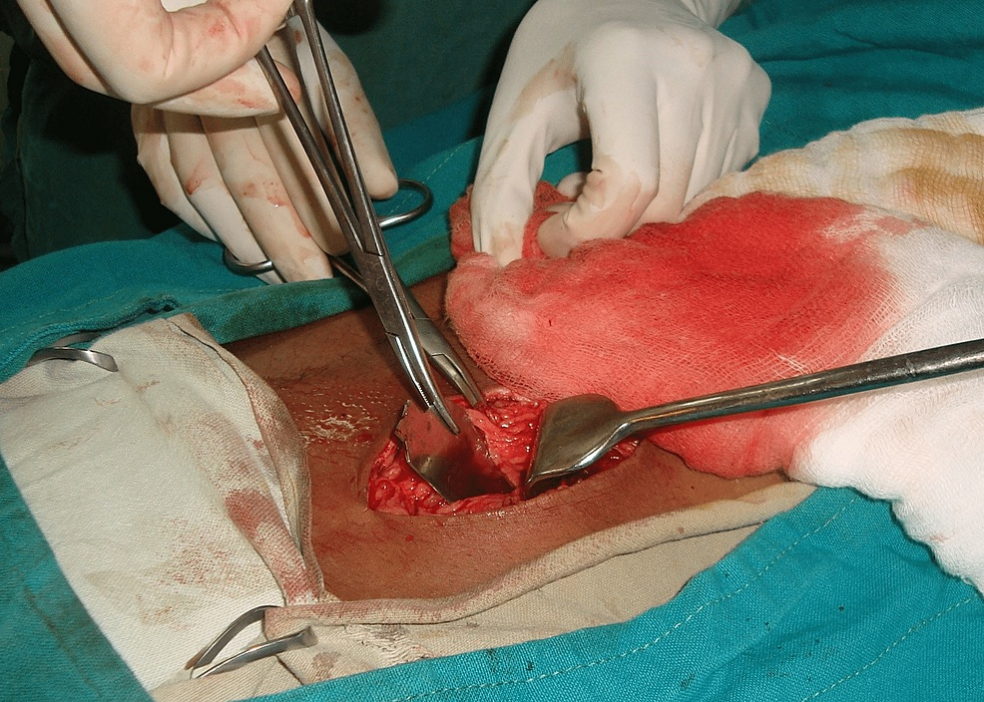
Retrieval of the knife blade from the abdominal cavity

**Figure 6:**
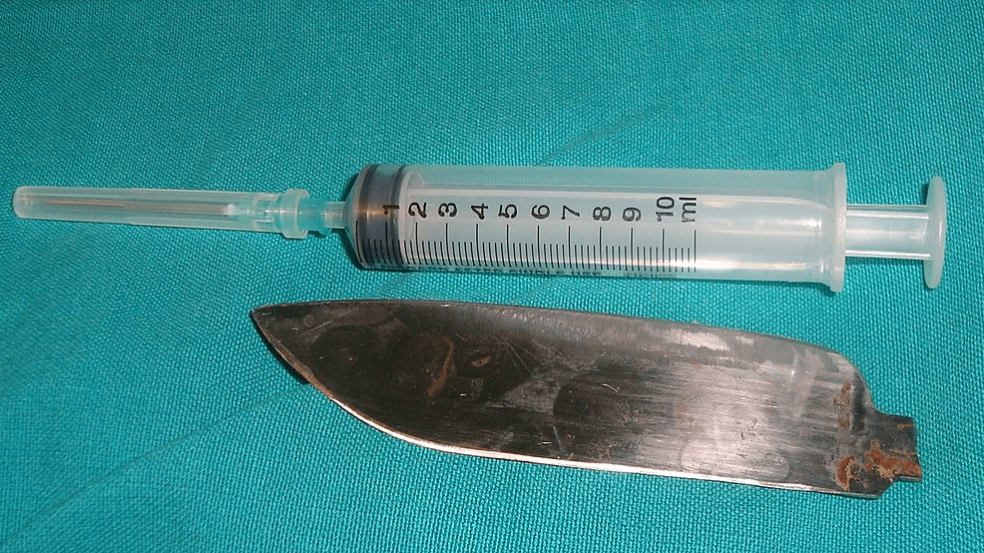
The retained knife blade after removal and comparison of size with a standard 10 ml syringe

He was taken to the operating room, and the imaging was repeated under fluoroscopy, which revealed the location of the knife blade inside the abdominal cavity (Figure 3). A laparotomy was performed, and a 15 cm long knife blade was found in his left iliac region. Fortunately, there was no severe injury to the surrounding organs. There was a 1 cm long superficial laceration on the anterior surface of the liver. The knife blade was wrapped by omentum (Figures 4-6). It had traveled from the right hypochondria to the left iliac region without injuring any viscera. Peritoneal washing revealed minimal blood but no intestinal contents. He made an uneventful postoperative recovery and was discharged on the fifth postoperative day. On the day of discharge, the patient was advised for a post-hospital follow-up in a week, and informed consent for the case report was taken. However, the patient did not arrive for follow-up.

## Discussion

Stab injuries to the abdomen and other parts of the body are common encounters we see in the hospital. However, the retained knife blade in the abdominal cavity is an uncommon occurrence. There have been only a few cases reporting retained knife blades in the body. In a retrospective chart review conducted by Sobnach et al., the most common site for retained knife blades was found to be the thorax, the least common site being the face and abdomen [2]. There are various reports of migrated ingested foreign bodies, the most common being clasp knives, blades, and needles [3].

The presentation of retained foreign bodies in the abdominal cavity may vary from being asymptomatic to having peritonitis. The presence of hemodynamic instability and peritonitis were high predictors of visceral injury leading to immediate laparotomy [4]. Our patient did not have these features and intraoperatively no major visceral injuries were identified.

All impacted knife injuries require careful clinical and radiological assessment. Clinical examination includes local wound exploration to determine the extent of abdominal wall injury [1]. It also helps assess whether the peritoneum has been violated or not. We think that proper wound exploration was not done at the previous center before suturing up the stab wound in the abdomen. Plain abdominal X-rays are routinely included in the diagnostic workup in most centers. In stable patients with abdominal and lower thoracic stabs, it helps to reveal possible extraluminal air in the peritoneal cavity [1,5]. However, there are relatively low positive and negative predictive values of extraluminal air in predicting the presence of a significant intra-abdominal organ injury [6]. After the plain X-ray revealed the retained knife, its presence was confirmed with the help of fluoroscopy. The next best imaging for penetrating abdominal injuries is contrast-enhanced computed tomography (CECT), which provides more information regarding visceral injuries and better localization of the knife blade inside the abdomen [4,7]. It was not available at our center, so we proceeded with fluoroscopy before performing laparotomy.

Management options in abdominal penetrating injuries vary from selective conservatism to laparotomy. Different trauma centers have different approaches to managing penetrating abdominal injuries, which are highly based on resource availability [8]. In every patient with a stab or open penetrating abdominal injury, examination of the peritoneum is most important. If the peritoneum is breached along with signs of peritonitis, the patient should be managed via exploratory laparotomy. If there is no sign of peritonitis, non-operative therapy is recommended [4,9]. Diagnostic peritoneal lavage (DPL) can be useful in determining the presence of a peritoneal breach in unclear cases [9]. CECT has important utility in identifying intra-abdominal injuries, which can guide the choice of management. If CECT does not identify intra-abdominal injury, diagnostic laparoscopy can be performed to assess the peritoneal breach, solid organ injury, and diaphragmatic injury, which can be further proceeded with therapeutic laparoscopy or laparotomy [7,9]. The patients who are selected for non-operative management should be observed for 48 hours in specialized centers with monitoring of vital signs, serial abdominal examination, hemoglobin monitoring, and low threshold to intervene if the clinical condition changes [7]. However, the clear-cut means of defining whether the patient needs operative or non-operative management in retained foreign bodies is less clear. Also, there is often a tendency to lean toward the side of caution, resulting in higher rates of laparotomy [9]. Our patient had a significantly large and sharp object in the abdominal cavity, which was very likely to have further complications like further organ injury and peritonitis, so the decision to perform a laparotomy was made. The knife blade was removed successfully, and no further management was required. In some cases, simple withdrawal can be performed safely in the emergency room provided potential life-threatening vascular and solid organ injuries have been excluded [2].

The retained knife blade is an unusual and spectacular injury. Retained stabbing instruments are seldom encountered in patients and have rarely been reported in the literature. In this case, the stab wound was located in the right hypochondrium and there was no information regarding the fate of the knife. The wondrous part was that the knife blade had traveled to the left iliac fossa without injuring any surrounding viscera. The potential hazard of having a sharp object freely moving in the abdominal cavity was significant with a high risk of additional injuries to organs and vessels. The superficial laceration in the liver in this patient was most likely caused by the knife after the initial assault. We also acknowledge that resource limitations at our center have affected our optimal approach to managing penetrating abdominal injury in our case. By the optimal utilization of available resources, we were able to manage this case with an uneventful recovery.

## Conclusions

In the evaluation of patients with abdominal stab wounds, attention should be given to the fate of the stabbing instruments, including retention inside the body. Potentially dangerous instruments can be retained in the abdominal cavity without causing significant symptoms. In unclear cases, plain X-rays are justified to detect or exclude a completely retained intra-abdominal object, which can cause additional injuries.
